# *N*-[1-(Biphenyl-4-yl)ethyl­idene]-*N*′-(2,4-dinitro­phen­yl)hydrazine

**DOI:** 10.1107/S1600536809009593

**Published:** 2009-04-08

**Authors:** Reza Kia, Hoong-Kun Fun, Bijan Etemadi, Hadi Kargar

**Affiliations:** a X-ray Crystallography Unit, School of Physics, Universiti Sains Malaysia, 11800 USM, Penang, Malaysia; b Department of Earth Sciences, College of Sciences, Shiraz University, Shiraz, Iran; c Department of Chemistry, School of Science, Payame Noor University (PNU), Ardakan, Yazd, Iran

## Abstract

The title compound, C_20_H_16_N_4_O_4_, contains two crystallographically independent mol­ecules (*A* and *B*) in the asymmetric unit. Intra­molecular N—H⋯O hydrogen bonds generate *S*(6) ring motifs in both molecules. The dihedral angles between the nitro-substituted benzene rings and the two  benzene rings in mol­ecules *A* and *B* are 14.32 (9), 17.89 (9)° and 13.04 (9) and 25.71 (9)°. The *ortho* and *para* nitro groups form dihedral angles of 6.2 (2) and 8.5 (2)° in mol­ecule *A*, and 5.3 (3) and 13.8 (2)° in mol­ecule *B*, with the benzene rings to which they are attached. The crystal structure is stabilized by inter­molecular C—H⋯O inter­actions.

## Related literature

For bond length data, see: Allen *et al.* (1987 ▶). For details of hydrogen-bond motifs, see: Bernstein *et al.* (1995 ▶). For general background and related structures, see: Fun *et al.* (2009 ▶); Kia *et al.* (2009 ▶); Cordis *et al.* (1998 ▶); Guillaumont & Nakamura (2000 ▶); Lamberton *et al.* (1974 ▶); Niknam *et al.* (2005 ▶); Raj & Kurup (2006 ▶); Zegota (1999 ▶); Zlotorzynska & Lai (1999 ▶); Okabe *et al.* (1993 ▶). For stability of the temperature controller used for data collection, see: Cosier & Glazer (1986 ▶).
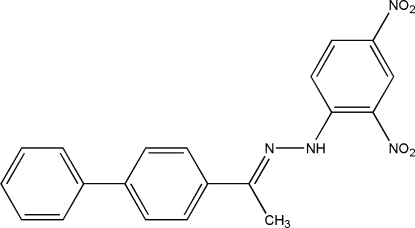


## Experimental

### 

#### Crystal data

C_20_H_16_N_4_O_4_
*M* *_r_* = 376.37
Monoclinic, 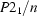

*a* = 10.0108 (5) Å
*b* = 14.9422 (8) Å
*c* = 23.3401 (14) Å
β = 99.871 (4)°
*V* = 3439.6 (3) Å^3^
*Z* = 8
Mo *K*α radiation
μ = 0.10 mm^−1^
*T* = 100 K
0.51 × 0.20 × 0.04 mm

#### Data collection

Bruker SMART APEXII CCD area-detector diffractometer
Absorption correction: multi-scan (*SADABS*; Bruker, 2005 ▶) *T*_min_ = 0.949, *T*_max_ = 0.996
38703 measured reflections
10018 independent reflections
6820 reflections with *I* > 2˘*I*)
*R*_int_ = 0.063

#### Refinement

*R*[*F* ^2^ > 2σ(*F* ^2^)] = 0.070
*wR*(*F* ^2^) = 0.162
*S* = 1.05
10018 reflections
515 parameters
H atoms treated by a mixture of independent and constrained refinement
Δρ_max_ = 0.35 e Å^−3^
Δρ_min_ = −0.32 e Å^−3^

### 

Data collection: *APEX2* (Bruker, 2005 ▶); cell refinement: *SAINT* (Bruker, 2005 ▶); data reduction: *SAINT*; program(s) used to solve structure: *SHELXTL* (Sheldrick, 2008 ▶); program(s) used to refine structure: *SHELXTL*; molecular graphics: *SHELXTL*; software used to prepare material for publication: *SHELXTL* and *PLATON* (Spek, 2009 ▶).

## Supplementary Material

Crystal structure: contains datablocks global, I. DOI: 10.1107/S1600536809009593/at2742sup1.cif

Structure factors: contains datablocks I. DOI: 10.1107/S1600536809009593/at2742Isup2.hkl

Additional supplementary materials:  crystallographic information; 3D view; checkCIF report

## Figures and Tables

**Table 1:** Hydrogen-bond geometry (Å, °)

*D*—H⋯*A*	*D*—H	H⋯*A*	*D*⋯*A*	*D*—H⋯*A*
N1*A*—H1N*A*⋯O1*A*	0.87 (3)	1.89 (2)	2.596 (2)	137 (2)
N1*B*—H1N*B*⋯O1*B*	0.78 (2)	1.99 (2)	2.600 (2)	134 (2)
C9*A*—H9*AA*⋯O2*A*^i^	0.95	2.41	3.084 (2)	127
C3*B*—H3*BA*⋯O1*B*^ii^	0.95	2.51	3.199 (2)	130
C9*B*—H9*BA*⋯O2*B*^ii^	0.95	2.39	3.174 (3)	139
C17*A*—H17*A*⋯O3*A*^iii^	0.95	2.59	3.498 (3)	161

Symmetry codes: (i) 
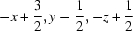
; (ii) 
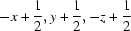
; (iii) 
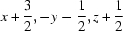
.

**Fig. 1.**
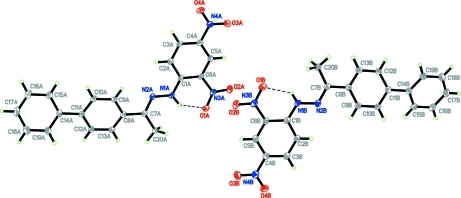
The molecular structure of the title compound, showing 50% probability displacement ellipsoids and the atomic numbering scheme. Hydrogen bond is shown as a dashed line.

**Fig. 2.**
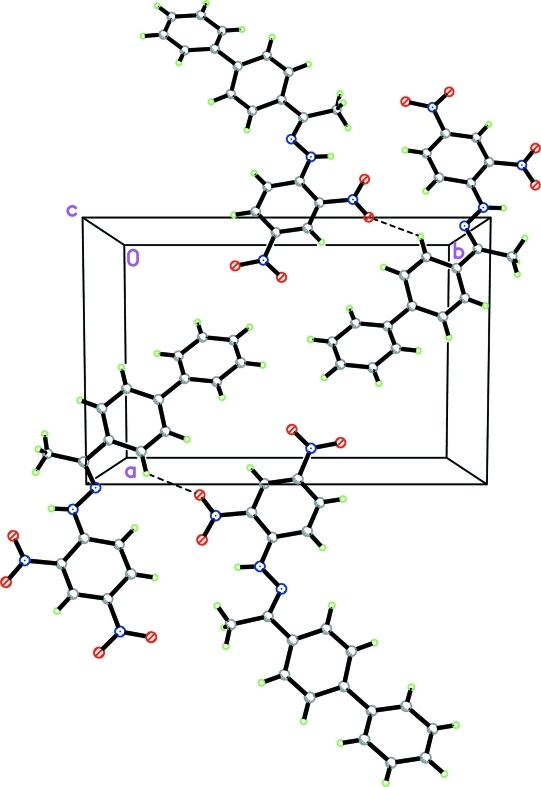
The crystal packing of the title compound, viewed down the c-axis, showing linking of molecules into dimers through intermolecular C—H···O interactions. Intermolecular interactions are shown as dashed lines.

## supplementary crystallographic information

### Comment

2,4-Dinitrophenylhydrazones play a more important role as stabilizers for the detection, characterization and protection of the carbonyl groups than phenylhydrazones (Niknam *et al.*, 2005). 2,4-Dinitrophenylhydrazone derivatives are widely used in various forms of analytical chemistry (Lamberton *et al.*, 1974; Zegota, 1999; Cordis *et al.*, 1998; Zlotorzynska & Lai, 1999) and are also used as dyes (Guillaumont & Nakamura, 2000). They are also found to have versatile coordinating abilities towards different metal ions (Raj & Kurup, 2006). In addition, some phenylhydrazone derivatives have been shown to be potentially DNA-damaging and mutagenic agents (Okabe *et al.*, 1993). For these reasons, the structure of the title compound was reported here.

The bond lengths (Allen *et al.*, 1987) and angles in the title compound (Fig. 1) have normal values and are comparable to the related structures (Fun *et al.* 2009; Kia *et al.* 2009). Intramolecular N—H···O hydrogen bonds generate *S(6)* ring motifs (Bernstein *et al.*,1995). The dihedral angle between the two benzene rings and the nitro-substituted benzene rings in molecules A and B are 14.32 (9), 17.89 (9), 13.04 (9), and 25.71 (9)°, respectively. The *ortho* and *para*-substituted nitro groups form dihedral angles of 6.2 (2), 8.5 (2)° in molecule A and 5.3 (3) and 13.8 (2)° in molecule B, to the benzene rings to which they are attached. The crystal structure is further stabilized by intermolecular C—H···O interactions (Table 1 and Fig.2 ).

### Experimental

The title compound was synthesized based on the reported procedure (Okabe *et al.* 1993) except that *p*-phenyl-acetophenone (1 mmol) was used instead. Single crystals suitable for X-ray diffraction analysis were grown by slow evaporation of a saturated solution of the resulted compound in acetone.

### Refinement

N-bound H atom was located from the difference Fourier map and refined freely; see, Table 1. The remaining H atoms were positioned geometrically and constrained with a riding model approximation with C—H = 0.95–0.98 Å and U_iso_(H) = 1.2 or 1.5 U_eq_(C). A rotating group model was applied to the methyl groups.

### Crystal data

**Table:**

C_20_H_16_N_4_O_4_	*F*(000) = 1568
*M**_r_* = 376.37	*D*_x_ = 1.454 Mg m^−^^3^
Monoclinic, *P*2_1_/*n*	Mo *K*α radiation, λ = 0.71073 Å
Hall symbol: -P 2yn	Cell parameters from 5139 reflections
*a* = 10.0108 (5) Å	θ = 2.5–28.5°
*b* = 14.9422 (8) Å	µ = 0.10 mm^−^^1^
*c* = 23.3401 (14) Å	*T* = 100 K
β = 99.871 (4)°	Plate, red
*V* = 3439.6 (3) Å^3^	0.51 × 0.20 × 0.04 mm
*Z* = 8	

### Data collection

**Table:**

Bruker SMART APEXII CCD area-detector diffractometer	10018 independent reflections
Radiation source: fine-focus sealed tube	6820 reflections with *I* > 2˘*I*)
graphite	*R*_int_ = 0.063
φ and ω scans	θ_max_ = 30.0°, θ_min_ = 1.6°
Absorption correction: multi-scan (*SADABS*; Bruker, 2005)	*h* = −14→14
*T*_min_ = 0.949, *T*_max_ = 0.996	*k* = −17→21
38703 measured reflections	*l* = −24→32

### Refinement

**Table:**

Refinement on *F*^2^	Primary atom site location: structure-invariant direct methods
Least-squares matrix: full	Secondary atom site location: difference Fourier map
*R*[*F*^2^ > 2σ(*F*^2^)] = 0.070	Hydrogen site location: inferred from neighbouring sites
*wR*(*F*^2^) = 0.162	H atoms treated by a mixture of independent and constrained refinement
*S* = 1.05	*w* = 1/[σ^2^(*F*_o_^2^) + (0.0656*P*)^2^ + 1.1266*P*] where *P* = (*F*_o_^2^ + 2*F*_c_^2^)/3
10018 reflections	(Δ/σ)_max_ < 0.001
515 parameters	Δρ_max_ = 0.35 e Å^−^^3^
0 restraints	Δρ_min_ = −0.32 e Å^−^^3^

### Special details

**Table:**

Experimental. The crystal was placed in the cold stream of an Oxford Cyrosystems Cobra open-flow nitrogen cryostat (Cosier & Glazer, 1986) operating at 100.0 (1)K.
Geometry. All esds (except the esd in the dihedral angle between two l.s. planes) are estimated using the full covariance matrix. The cell esds are taken into account individually in the estimation of esds in distances, angles and torsion angles; correlations between esds in cell parameters are only used when they are defined by crystal symmetry. An approximate (isotropic) treatment of cell esds is used for estimating esds involving l.s. planes.
Refinement. Refinement of F^2^ against ALL reflections. The weighted R-factor wR and goodness of fit S are based on F^2^, conventional R-factors R are based on F, with F set to zero for negative F^2^. The threshold expression of F^2^ > 2sigma(F^2^) is used only for calculating R-factors(gt) etc. and is not relevant to the choice of reflections for refinement. R-factors based on F^2^ are statistically about twice as large as those based on F, and R- factors based on ALL data will be even larger.

### Fractional atomic coordinates and isotropic or equivalent isotropic displacement parameters (Å^2^)

**Table:**

	*x*	*y*	*z*	*U*_iso_*/*U*_eq_	
O1A	0.76069 (14)	0.22620 (9)	0.23590 (6)	0.0246 (3)	
O2A	0.58648 (18)	0.23537 (10)	0.16763 (8)	0.0485 (5)	
O3A	0.32139 (14)	−0.01433 (9)	0.09644 (7)	0.0278 (3)	
O4A	0.37325 (14)	−0.14680 (9)	0.13206 (7)	0.0280 (3)	
N1A	0.86347 (15)	0.07099 (11)	0.26819 (7)	0.0172 (3)	
H1NA	0.866 (2)	0.1289 (17)	0.2702 (11)	0.039 (7)*	
N2A	0.94751 (15)	0.01368 (10)	0.30306 (7)	0.0171 (3)	
N3A	0.66711 (17)	0.19105 (10)	0.20141 (8)	0.0243 (4)	
N4A	0.39718 (16)	−0.06641 (11)	0.12752 (7)	0.0209 (3)	
C1A	0.75102 (18)	0.03800 (12)	0.23386 (8)	0.0163 (4)	
C2A	0.72659 (18)	−0.05521 (12)	0.22963 (8)	0.0185 (4)	
H2AA	0.7896	−0.0951	0.2515	0.022*	
C3A	0.61413 (19)	−0.08917 (12)	0.19472 (8)	0.0196 (4)	
H3AA	0.5996	−0.1520	0.1924	0.024*	
C4A	0.52048 (18)	−0.03081 (12)	0.16247 (8)	0.0189 (4)	
C5A	0.53958 (18)	0.06021 (12)	0.16430 (8)	0.0195 (4)	
H5AA	0.4764	0.0990	0.1415	0.023*	
C6A	0.65363 (18)	0.09453 (12)	0.20027 (9)	0.0187 (4)	
C7A	1.05071 (18)	0.04897 (12)	0.33658 (8)	0.0172 (4)	
C8A	1.13686 (18)	−0.01546 (12)	0.37413 (8)	0.0173 (4)	
C9A	1.08960 (19)	−0.10274 (12)	0.37967 (8)	0.0200 (4)	
H9AA	1.0000	−0.1179	0.3617	0.024*	
C10A	1.17056 (19)	−0.16714 (12)	0.41074 (9)	0.0203 (4)	
H10A	1.1364	−0.2261	0.4132	0.024*	
C11A	1.30261 (18)	−0.14688 (12)	0.43877 (8)	0.0180 (4)	
C12A	1.34820 (19)	−0.05927 (12)	0.43486 (8)	0.0196 (4)	
H12A	1.4364	−0.0436	0.4542	0.024*	
C13A	1.26710 (18)	0.00583 (12)	0.40313 (8)	0.0186 (4)	
H13A	1.3006	0.0650	0.4012	0.022*	
C14A	1.38924 (18)	−0.21713 (12)	0.47113 (8)	0.0194 (4)	
C15A	1.3764 (2)	−0.30658 (13)	0.45349 (9)	0.0234 (4)	
H15A	1.3121	−0.3224	0.4202	0.028*	
C16A	1.4563 (2)	−0.37248 (14)	0.48411 (10)	0.0277 (5)	
H16A	1.4457	−0.4330	0.4718	0.033*	
C17A	1.5511 (2)	−0.35083 (14)	0.53224 (10)	0.0287 (5)	
H17A	1.6069	−0.3960	0.5525	0.034*	
C18A	1.5644 (2)	−0.26259 (14)	0.55081 (10)	0.0297 (5)	
H18A	1.6286	−0.2474	0.5843	0.036*	
C19A	1.4841 (2)	−0.19649 (14)	0.52056 (9)	0.0246 (4)	
H19A	1.4938	−0.1363	0.5336	0.029*	
C20A	1.08413 (19)	0.14713 (12)	0.33820 (9)	0.0214 (4)	
H20A	1.0061	0.1814	0.3464	0.032*	
H20B	1.1055	0.1656	0.3005	0.032*	
H20C	1.1625	0.1584	0.3687	0.032*	
O1B	0.24703 (15)	0.26357 (9)	0.23255 (7)	0.0323 (4)	
O2B	0.4466 (2)	0.24962 (11)	0.28413 (10)	0.0599 (6)	
O3B	0.66656 (15)	0.48817 (10)	0.39824 (7)	0.0307 (3)	
O4B	0.60838 (14)	0.62418 (9)	0.37192 (6)	0.0278 (3)	
N1B	0.13039 (16)	0.41932 (11)	0.21771 (8)	0.0203 (4)	
H1NB	0.126 (2)	0.3682 (15)	0.2101 (10)	0.025 (6)*	
N2B	0.03856 (15)	0.48109 (10)	0.19137 (7)	0.0189 (3)	
N3B	0.34763 (19)	0.29565 (11)	0.26485 (9)	0.0306 (4)	
N4B	0.58899 (16)	0.54278 (11)	0.37022 (7)	0.0230 (4)	
C1B	0.24162 (18)	0.44779 (12)	0.25439 (8)	0.0184 (4)	
C2B	0.25660 (19)	0.53943 (12)	0.27013 (9)	0.0200 (4)	
H2BA	0.1880	0.5808	0.2543	0.024*	
C3B	0.36773 (19)	0.56955 (13)	0.30758 (9)	0.0210 (4)	
H3BA	0.3763	0.6313	0.3171	0.025*	
C4B	0.46877 (19)	0.50923 (12)	0.33182 (8)	0.0198 (4)	
C5B	0.46021 (19)	0.41996 (13)	0.31842 (9)	0.0215 (4)	
H5BA	0.5293	0.3797	0.3354	0.026*	
C6B	0.34862 (19)	0.38920 (12)	0.27952 (9)	0.0206 (4)	
C7B	−0.06623 (19)	0.45005 (12)	0.15687 (8)	0.0190 (4)	
C8B	−0.15944 (18)	0.51924 (12)	0.12712 (8)	0.0186 (4)	
C9B	−0.14824 (19)	0.60798 (12)	0.14622 (8)	0.0191 (4)	
H9BA	−0.0812	0.6235	0.1786	0.023*	
C10B	−0.23311 (18)	0.67353 (12)	0.11872 (8)	0.0191 (4)	
H10B	−0.2242	0.7332	0.1330	0.023*	
C11B	−0.33207 (18)	0.65396 (12)	0.07024 (9)	0.0190 (4)	
C12B	−0.34157 (19)	0.56507 (13)	0.05072 (9)	0.0214 (4)	
H12B	−0.4066	0.5498	0.0175	0.026*	
C13B	−0.25785 (19)	0.49892 (13)	0.07897 (9)	0.0207 (4)	
H13B	−0.2677	0.4390	0.0653	0.025*	
C14B	−0.42078 (19)	0.72507 (13)	0.03980 (8)	0.0198 (4)	
C15B	−0.3711 (2)	0.81216 (13)	0.03623 (9)	0.0228 (4)	
H15B	−0.2814	0.8263	0.0547	0.027*	
C16B	−0.4522 (2)	0.87809 (14)	0.00587 (9)	0.0272 (5)	
H16B	−0.4173	0.9368	0.0033	0.033*	
C17B	−0.5834 (2)	0.85851 (14)	−0.02069 (9)	0.0289 (5)	
H17B	−0.6381	0.9037	−0.0416	0.035*	
C18B	−0.6353 (2)	0.77304 (14)	−0.01678 (9)	0.0280 (5)	
H18B	−0.7258	0.7598	−0.0345	0.034*	
C19B	−0.5540 (2)	0.70702 (14)	0.01314 (9)	0.0235 (4)	
H19B	−0.5897	0.6485	0.0155	0.028*	
C20B	−0.0951 (2)	0.35244 (12)	0.14521 (9)	0.0233 (4)	
H20D	−0.0688	0.3184	0.1813	0.035*	
H20E	−0.0432	0.3311	0.1159	0.035*	
H20F	−0.1922	0.3441	0.1308	0.035*	

### Atomic displacement parameters (Å^2^)

**Table:**

	*U*^11^	*U*^22^	*U*^33^	*U*^12^	*U*^13^	*U*^23^
O1A	0.0273 (7)	0.0135 (7)	0.0311 (8)	−0.0023 (5)	−0.0008 (6)	−0.0008 (6)
O2A	0.0494 (10)	0.0149 (8)	0.0677 (13)	0.0038 (7)	−0.0279 (9)	0.0072 (8)
O3A	0.0255 (7)	0.0247 (8)	0.0305 (8)	0.0004 (6)	−0.0033 (6)	0.0005 (6)
O4A	0.0298 (8)	0.0167 (7)	0.0364 (9)	−0.0058 (6)	0.0028 (7)	−0.0039 (6)
N1A	0.0191 (8)	0.0100 (7)	0.0219 (9)	0.0013 (6)	0.0019 (6)	0.0020 (6)
N2A	0.0189 (7)	0.0132 (7)	0.0193 (8)	0.0021 (6)	0.0034 (6)	0.0031 (6)
N3A	0.0276 (9)	0.0115 (8)	0.0315 (10)	0.0014 (6)	−0.0011 (7)	0.0008 (7)
N4A	0.0216 (8)	0.0182 (8)	0.0229 (9)	−0.0009 (6)	0.0037 (7)	−0.0037 (7)
C1A	0.0184 (8)	0.0126 (8)	0.0189 (10)	0.0008 (7)	0.0061 (7)	0.0008 (7)
C2A	0.0210 (9)	0.0137 (9)	0.0208 (10)	0.0009 (7)	0.0040 (7)	0.0017 (7)
C3A	0.0233 (9)	0.0115 (9)	0.0246 (10)	−0.0007 (7)	0.0056 (8)	0.0000 (7)
C4A	0.0194 (9)	0.0171 (9)	0.0203 (10)	−0.0012 (7)	0.0039 (7)	−0.0011 (7)
C5A	0.0193 (9)	0.0159 (9)	0.0230 (10)	0.0022 (7)	0.0033 (7)	0.0014 (8)
C6A	0.0198 (9)	0.0113 (8)	0.0250 (10)	0.0007 (7)	0.0041 (7)	0.0013 (7)
C7A	0.0193 (9)	0.0141 (9)	0.0190 (10)	−0.0009 (7)	0.0052 (7)	−0.0006 (7)
C8A	0.0198 (9)	0.0130 (9)	0.0195 (10)	0.0009 (7)	0.0044 (7)	−0.0007 (7)
C9A	0.0198 (9)	0.0149 (9)	0.0239 (10)	−0.0021 (7)	−0.0002 (7)	−0.0021 (8)
C10A	0.0232 (9)	0.0129 (9)	0.0242 (10)	−0.0005 (7)	0.0025 (8)	0.0016 (8)
C11A	0.0199 (9)	0.0163 (9)	0.0182 (10)	0.0013 (7)	0.0046 (7)	−0.0006 (7)
C12A	0.0182 (9)	0.0180 (9)	0.0224 (10)	−0.0012 (7)	0.0029 (7)	−0.0011 (8)
C13A	0.0209 (9)	0.0135 (9)	0.0216 (10)	−0.0020 (7)	0.0047 (7)	−0.0002 (7)
C14A	0.0181 (9)	0.0201 (10)	0.0209 (10)	0.0023 (7)	0.0058 (7)	0.0050 (8)
C15A	0.0267 (10)	0.0196 (10)	0.0242 (11)	0.0022 (8)	0.0051 (8)	0.0012 (8)
C16A	0.0327 (11)	0.0197 (10)	0.0323 (12)	0.0071 (8)	0.0097 (9)	0.0043 (9)
C17A	0.0229 (10)	0.0283 (11)	0.0355 (13)	0.0079 (8)	0.0065 (9)	0.0127 (10)
C18A	0.0247 (10)	0.0322 (12)	0.0301 (12)	−0.0008 (9)	−0.0011 (9)	0.0059 (10)
C19A	0.0228 (10)	0.0215 (10)	0.0283 (11)	0.0005 (8)	0.0011 (8)	0.0042 (8)
C20A	0.0232 (9)	0.0127 (9)	0.0267 (11)	−0.0019 (7)	−0.0007 (8)	0.0003 (8)
O1B	0.0347 (8)	0.0152 (7)	0.0431 (10)	−0.0039 (6)	−0.0040 (7)	0.0001 (7)
O2B	0.0561 (12)	0.0208 (9)	0.0871 (16)	0.0157 (8)	−0.0319 (11)	−0.0047 (9)
O3B	0.0257 (7)	0.0305 (8)	0.0330 (9)	0.0016 (6)	−0.0027 (6)	0.0051 (7)
O4B	0.0290 (8)	0.0211 (8)	0.0334 (9)	−0.0027 (6)	0.0059 (6)	−0.0048 (6)
N1B	0.0223 (8)	0.0094 (8)	0.0286 (10)	−0.0005 (6)	0.0030 (7)	0.0023 (7)
N2B	0.0194 (8)	0.0153 (8)	0.0223 (9)	0.0014 (6)	0.0046 (6)	0.0033 (6)
N3B	0.0334 (10)	0.0144 (8)	0.0405 (11)	0.0011 (7)	−0.0033 (8)	0.0031 (8)
N4B	0.0213 (8)	0.0236 (9)	0.0253 (9)	0.0000 (7)	0.0075 (7)	−0.0006 (7)
C1B	0.0199 (9)	0.0156 (9)	0.0209 (10)	−0.0009 (7)	0.0070 (7)	0.0024 (7)
C2B	0.0215 (9)	0.0149 (9)	0.0247 (10)	0.0012 (7)	0.0068 (8)	0.0020 (8)
C3B	0.0239 (9)	0.0153 (9)	0.0251 (11)	0.0002 (7)	0.0079 (8)	0.0013 (8)
C4B	0.0212 (9)	0.0182 (9)	0.0206 (10)	−0.0019 (7)	0.0054 (7)	0.0018 (8)
C5B	0.0197 (9)	0.0216 (10)	0.0233 (10)	0.0017 (7)	0.0039 (8)	0.0049 (8)
C6B	0.0238 (10)	0.0121 (9)	0.0264 (11)	0.0005 (7)	0.0057 (8)	0.0033 (8)
C7B	0.0217 (9)	0.0162 (9)	0.0209 (10)	−0.0010 (7)	0.0083 (8)	0.0012 (8)
C8B	0.0179 (9)	0.0171 (9)	0.0214 (10)	−0.0006 (7)	0.0052 (7)	0.0017 (8)
C9B	0.0200 (9)	0.0163 (9)	0.0209 (10)	−0.0030 (7)	0.0032 (7)	0.0002 (8)
C10B	0.0211 (9)	0.0145 (9)	0.0220 (10)	−0.0010 (7)	0.0043 (8)	−0.0017 (7)
C11B	0.0184 (9)	0.0181 (9)	0.0216 (10)	0.0000 (7)	0.0065 (7)	0.0023 (8)
C12B	0.0197 (9)	0.0205 (10)	0.0230 (10)	−0.0030 (7)	0.0005 (8)	0.0000 (8)
C13B	0.0227 (9)	0.0150 (9)	0.0242 (10)	−0.0043 (7)	0.0041 (8)	−0.0028 (8)
C14B	0.0216 (9)	0.0203 (10)	0.0180 (10)	0.0015 (7)	0.0051 (7)	−0.0008 (8)
C15B	0.0251 (10)	0.0204 (10)	0.0230 (11)	0.0011 (7)	0.0044 (8)	−0.0012 (8)
C16B	0.0353 (12)	0.0180 (10)	0.0292 (12)	0.0034 (8)	0.0079 (9)	0.0016 (9)
C17B	0.0329 (11)	0.0279 (11)	0.0248 (11)	0.0114 (9)	0.0021 (9)	0.0019 (9)
C18B	0.0238 (10)	0.0335 (12)	0.0259 (11)	0.0043 (8)	0.0018 (8)	−0.0033 (9)
C19B	0.0243 (10)	0.0237 (10)	0.0228 (10)	0.0000 (8)	0.0045 (8)	−0.0014 (8)
C20B	0.0255 (10)	0.0137 (9)	0.0310 (11)	−0.0029 (7)	0.0053 (8)	0.0009 (8)

### Geometric parameters (Å, °)

**Table:**

O1A—N3A	1.242 (2)	O1B—N3B	1.246 (2)
O2A—N3A	1.222 (2)	O2B—N3B	1.226 (2)
O3A—N4A	1.232 (2)	O3B—N4B	1.234 (2)
O4A—N4A	1.233 (2)	O4B—N4B	1.231 (2)
N1A—C1A	1.358 (2)	N1B—C1B	1.352 (2)
N1A—N2A	1.367 (2)	N1B—N2B	1.372 (2)
N1A—H1NA	0.87 (2)	N1B—H1NB	0.78 (2)
N2A—C7A	1.296 (2)	N2B—C7B	1.294 (2)
N3A—C6A	1.448 (2)	N3B—C6B	1.439 (2)
N4A—C4A	1.458 (2)	N4B—C4B	1.461 (3)
C1A—C2A	1.415 (2)	C1B—C2B	1.419 (3)
C1A—C6A	1.420 (2)	C1B—C6B	1.429 (3)
C2A—C3A	1.369 (3)	C2B—C3B	1.368 (3)
C2A—H2AA	0.9500	C2B—H2BA	0.9500
C3A—C4A	1.401 (3)	C3B—C4B	1.400 (3)
C3A—H3AA	0.9500	C3B—H3BA	0.9500
C4A—C5A	1.373 (3)	C4B—C5B	1.369 (3)
C5A—C6A	1.394 (3)	C5B—C6B	1.391 (3)
C5A—H5AA	0.9500	C5B—H5BA	0.9500
C7A—C8A	1.476 (3)	C7B—C8B	1.483 (3)
C7A—C20A	1.504 (2)	C7B—C20B	1.503 (3)
C8A—C13A	1.399 (3)	C8B—C13B	1.395 (3)
C8A—C9A	1.401 (2)	C8B—C9B	1.397 (3)
C9A—C10A	1.381 (3)	C9B—C10B	1.381 (3)
C9A—H9AA	0.9500	C9B—H9BA	0.9500
C10A—C11A	1.404 (3)	C10B—C11B	1.401 (3)
C10A—H10A	0.9500	C10B—H10B	0.9500
C11A—C12A	1.394 (3)	C11B—C12B	1.402 (3)
C11A—C14A	1.483 (3)	C11B—C14B	1.485 (3)
C12A—C13A	1.395 (3)	C12B—C13B	1.387 (3)
C12A—H12A	0.9500	C12B—H12B	0.9500
C13A—H13A	0.9500	C13B—H13B	0.9500
C14A—C19A	1.397 (3)	C14B—C19B	1.398 (3)
C14A—C15A	1.398 (3)	C14B—C15B	1.401 (3)
C15A—C16A	1.387 (3)	C15B—C16B	1.391 (3)
C15A—H15A	0.9500	C15B—H15B	0.9500
C16A—C17A	1.379 (3)	C16B—C17B	1.385 (3)
C16A—H16A	0.9500	C16B—H16B	0.9500
C17A—C18A	1.387 (3)	C17B—C18B	1.387 (3)
C17A—H17A	0.9500	C17B—H17B	0.9500
C18A—C19A	1.388 (3)	C18B—C19B	1.388 (3)
C18A—H18A	0.9500	C18B—H18B	0.9500
C19A—H19A	0.9500	C19B—H19B	0.9500
C20A—H20A	0.9800	C20B—H20D	0.9800
C20A—H20B	0.9800	C20B—H20E	0.9800
C20A—H20C	0.9800	C20B—H20F	0.9800
			
C1A—N1A—N2A	119.17 (15)	C1B—N1B—N2B	119.21 (16)
C1A—N1A—H1NA	114.3 (17)	C1B—N1B—H1NB	117.1 (17)
N2A—N1A—H1NA	125.6 (17)	N2B—N1B—H1NB	123.4 (17)
C7A—N2A—N1A	116.74 (15)	C7B—N2B—N1B	116.56 (16)
O2A—N3A—O1A	122.02 (16)	O2B—N3B—O1B	121.65 (18)
O2A—N3A—C6A	118.59 (17)	O2B—N3B—C6B	119.03 (18)
O1A—N3A—C6A	119.38 (16)	O1B—N3B—C6B	119.32 (17)
O3A—N4A—O4A	123.82 (16)	O4B—N4B—O3B	123.70 (17)
O3A—N4A—C4A	118.40 (15)	O4B—N4B—C4B	117.88 (16)
O4A—N4A—C4A	117.75 (16)	O3B—N4B—C4B	118.41 (16)
N1A—C1A—C2A	120.97 (17)	N1B—C1B—C2B	120.45 (17)
N1A—C1A—C6A	122.09 (16)	N1B—C1B—C6B	123.04 (17)
C2A—C1A—C6A	116.93 (17)	C2B—C1B—C6B	116.52 (17)
C3A—C2A—C1A	121.40 (17)	C3B—C2B—C1B	121.41 (18)
C3A—C2A—H2AA	119.3	C3B—C2B—H2BA	119.3
C1A—C2A—H2AA	119.3	C1B—C2B—H2BA	119.3
C2A—C3A—C4A	119.68 (17)	C2B—C3B—C4B	119.92 (18)
C2A—C3A—H3AA	120.2	C2B—C3B—H3BA	120.0
C4A—C3A—H3AA	120.2	C4B—C3B—H3BA	120.0
C5A—C4A—C3A	121.53 (18)	C5B—C4B—C3B	121.48 (18)
C5A—C4A—N4A	118.60 (17)	C5B—C4B—N4B	119.16 (17)
C3A—C4A—N4A	119.84 (16)	C3B—C4B—N4B	119.30 (17)
C4A—C5A—C6A	118.58 (17)	C4B—C5B—C6B	118.86 (18)
C4A—C5A—H5AA	120.7	C4B—C5B—H5BA	120.6
C6A—C5A—H5AA	120.7	C6B—C5B—H5BA	120.6
C5A—C6A—C1A	121.85 (16)	C5B—C6B—C1B	121.79 (17)
C5A—C6A—N3A	116.20 (16)	C5B—C6B—N3B	116.63 (17)
C1A—C6A—N3A	121.94 (16)	C1B—C6B—N3B	121.56 (18)
N2A—C7A—C8A	114.65 (16)	N2B—C7B—C8B	114.79 (16)
N2A—C7A—C20A	124.26 (17)	N2B—C7B—C20B	124.82 (18)
C8A—C7A—C20A	121.09 (16)	C8B—C7B—C20B	120.38 (17)
C13A—C8A—C9A	117.93 (17)	C13B—C8B—C9B	118.07 (17)
C13A—C8A—C7A	122.70 (16)	C13B—C8B—C7B	121.75 (17)
C9A—C8A—C7A	119.32 (16)	C9B—C8B—C7B	120.16 (17)
C10A—C9A—C8A	121.34 (18)	C10B—C9B—C8B	121.04 (18)
C10A—C9A—H9AA	119.3	C10B—C9B—H9BA	119.5
C8A—C9A—H9AA	119.3	C8B—C9B—H9BA	119.5
C9A—C10A—C11A	121.02 (17)	C9B—C10B—C11B	121.39 (17)
C9A—C10A—H10A	119.5	C9B—C10B—H10B	119.3
C11A—C10A—H10A	119.5	C11B—C10B—H10B	119.3
C12A—C11A—C10A	117.69 (17)	C10B—C11B—C12B	117.35 (17)
C12A—C11A—C14A	121.89 (17)	C10B—C11B—C14B	121.27 (17)
C10A—C11A—C14A	120.42 (16)	C12B—C11B—C14B	121.37 (18)
C11A—C12A—C13A	121.46 (17)	C13B—C12B—C11B	121.24 (18)
C11A—C12A—H12A	119.3	C13B—C12B—H12B	119.4
C13A—C12A—H12A	119.3	C11B—C12B—H12B	119.4
C12A—C13A—C8A	120.50 (17)	C12B—C13B—C8B	120.90 (18)
C12A—C13A—H13A	119.8	C12B—C13B—H13B	119.6
C8A—C13A—H13A	119.8	C8B—C13B—H13B	119.6
C19A—C14A—C15A	118.00 (18)	C19B—C14B—C15B	118.31 (18)
C19A—C14A—C11A	121.30 (17)	C19B—C14B—C11B	121.43 (17)
C15A—C14A—C11A	120.69 (18)	C15B—C14B—C11B	120.25 (17)
C16A—C15A—C14A	120.7 (2)	C16B—C15B—C14B	120.41 (19)
C16A—C15A—H15A	119.6	C16B—C15B—H15B	119.8
C14A—C15A—H15A	119.6	C14B—C15B—H15B	119.8
C17A—C16A—C15A	120.6 (2)	C17B—C16B—C15B	120.31 (19)
C17A—C16A—H16A	119.7	C17B—C16B—H16B	119.8
C15A—C16A—H16A	119.7	C15B—C16B—H16B	119.8
C16A—C17A—C18A	119.57 (19)	C16B—C17B—C18B	120.1 (2)
C16A—C17A—H17A	120.2	C16B—C17B—H17B	119.9
C18A—C17A—H17A	120.2	C18B—C17B—H17B	119.9
C17A—C18A—C19A	120.1 (2)	C17B—C18B—C19B	119.6 (2)
C17A—C18A—H18A	120.0	C17B—C18B—H18B	120.2
C19A—C18A—H18A	120.0	C19B—C18B—H18B	120.2
C18A—C19A—C14A	121.0 (2)	C18B—C19B—C14B	121.25 (19)
C18A—C19A—H19A	119.5	C18B—C19B—H19B	119.4
C14A—C19A—H19A	119.5	C14B—C19B—H19B	119.4
C7A—C20A—H20A	109.5	C7B—C20B—H20D	109.5
C7A—C20A—H20B	109.5	C7B—C20B—H20E	109.5
H20A—C20A—H20B	109.5	H20D—C20B—H20E	109.5
C7A—C20A—H20C	109.5	C7B—C20B—H20F	109.5
H20A—C20A—H20C	109.5	H20D—C20B—H20F	109.5
H20B—C20A—H20C	109.5	H20E—C20B—H20F	109.5
			
C1A—N1A—N2A—C7A	−177.50 (16)	C1B—N1B—N2B—C7B	−179.75 (16)
N2A—N1A—C1A—C2A	−6.0 (2)	N2B—N1B—C1B—C2B	6.4 (3)
N2A—N1A—C1A—C6A	174.68 (16)	N2B—N1B—C1B—C6B	−174.03 (17)
N1A—C1A—C2A—C3A	−179.39 (17)	N1B—C1B—C2B—C3B	179.40 (17)
C6A—C1A—C2A—C3A	0.0 (3)	C6B—C1B—C2B—C3B	−0.2 (3)
C1A—C2A—C3A—C4A	−0.2 (3)	C1B—C2B—C3B—C4B	−0.8 (3)
C2A—C3A—C4A—C5A	1.0 (3)	C2B—C3B—C4B—C5B	0.8 (3)
C2A—C3A—C4A—N4A	−176.91 (16)	C2B—C3B—C4B—N4B	177.97 (16)
O3A—N4A—C4A—C5A	7.1 (3)	O4B—N4B—C4B—C5B	164.84 (17)
O4A—N4A—C4A—C5A	−171.05 (17)	O3B—N4B—C4B—C5B	−13.8 (3)
O3A—N4A—C4A—C3A	−174.96 (17)	O4B—N4B—C4B—C3B	−12.4 (2)
O4A—N4A—C4A—C3A	6.9 (3)	O3B—N4B—C4B—C3B	168.92 (17)
C3A—C4A—C5A—C6A	−1.5 (3)	C3B—C4B—C5B—C6B	0.4 (3)
N4A—C4A—C5A—C6A	176.43 (16)	N4B—C4B—C5B—C6B	−176.81 (16)
C4A—C5A—C6A—C1A	1.3 (3)	C4B—C5B—C6B—C1B	−1.5 (3)
C4A—C5A—C6A—N3A	−178.73 (17)	C4B—C5B—C6B—N3B	177.20 (17)
N1A—C1A—C6A—C5A	178.84 (17)	N1B—C1B—C6B—C5B	−178.20 (18)
C2A—C1A—C6A—C5A	−0.5 (3)	C2B—C1B—C6B—C5B	1.4 (3)
N1A—C1A—C6A—N3A	−1.2 (3)	N1B—C1B—C6B—N3B	3.2 (3)
C2A—C1A—C6A—N3A	179.46 (16)	C2B—C1B—C6B—N3B	−177.22 (17)
O2A—N3A—C6A—C5A	−6.5 (3)	O2B—N3B—C6B—C5B	−4.2 (3)
O1A—N3A—C6A—C5A	174.46 (17)	O1B—N3B—C6B—C5B	176.37 (18)
O2A—N3A—C6A—C1A	173.51 (19)	O2B—N3B—C6B—C1B	174.5 (2)
O1A—N3A—C6A—C1A	−5.5 (3)	O1B—N3B—C6B—C1B	−4.9 (3)
N1A—N2A—C7A—C8A	178.93 (15)	N1B—N2B—C7B—C8B	−177.16 (15)
N1A—N2A—C7A—C20A	−1.4 (3)	N1B—N2B—C7B—C20B	1.7 (3)
N2A—C7A—C8A—C13A	165.23 (17)	N2B—C7B—C8B—C13B	164.50 (17)
C20A—C7A—C8A—C13A	−14.4 (3)	C20B—C7B—C8B—C13B	−14.4 (3)
N2A—C7A—C8A—C9A	−12.4 (2)	N2B—C7B—C8B—C9B	−14.0 (2)
C20A—C7A—C8A—C9A	167.97 (17)	C20B—C7B—C8B—C9B	167.14 (17)
C13A—C8A—C9A—C10A	−2.7 (3)	C13B—C8B—C9B—C10B	0.8 (3)
C7A—C8A—C9A—C10A	175.07 (17)	C7B—C8B—C9B—C10B	179.31 (17)
C8A—C9A—C10A—C11A	1.1 (3)	C8B—C9B—C10B—C11B	−1.0 (3)
C9A—C10A—C11A—C12A	1.0 (3)	C9B—C10B—C11B—C12B	0.0 (3)
C9A—C10A—C11A—C14A	−178.94 (17)	C9B—C10B—C11B—C14B	−178.35 (17)
C10A—C11A—C12A—C13A	−1.5 (3)	C10B—C11B—C12B—C13B	1.1 (3)
C14A—C11A—C12A—C13A	178.42 (17)	C14B—C11B—C12B—C13B	179.48 (17)
C11A—C12A—C13A—C8A	−0.1 (3)	C11B—C12B—C13B—C8B	−1.3 (3)
C9A—C8A—C13A—C12A	2.1 (3)	C9B—C8B—C13B—C12B	0.4 (3)
C7A—C8A—C13A—C12A	−175.51 (17)	C7B—C8B—C13B—C12B	−178.16 (17)
C12A—C11A—C14A—C19A	32.5 (3)	C10B—C11B—C14B—C19B	−147.87 (18)
C10A—C11A—C14A—C19A	−147.56 (19)	C12B—C11B—C14B—C19B	33.8 (3)
C12A—C11A—C14A—C15A	−148.62 (18)	C10B—C11B—C14B—C15B	33.4 (3)
C10A—C11A—C14A—C15A	31.3 (3)	C12B—C11B—C14B—C15B	−144.91 (18)
C19A—C14A—C15A—C16A	−0.4 (3)	C19B—C14B—C15B—C16B	−1.2 (3)
C11A—C14A—C15A—C16A	−179.29 (17)	C11B—C14B—C15B—C16B	177.55 (18)
C14A—C15A—C16A—C17A	−0.7 (3)	C14B—C15B—C16B—C17B	0.6 (3)
C15A—C16A—C17A—C18A	1.3 (3)	C15B—C16B—C17B—C18B	0.5 (3)
C16A—C17A—C18A—C19A	−0.9 (3)	C16B—C17B—C18B—C19B	−1.0 (3)
C17A—C18A—C19A—C14A	−0.1 (3)	C17B—C18B—C19B—C14B	0.4 (3)
C15A—C14A—C19A—C18A	0.8 (3)	C15B—C14B—C19B—C18B	0.7 (3)
C11A—C14A—C19A—C18A	179.68 (18)	C11B—C14B—C19B—C18B	−178.02 (18)

### Hydrogen-bond geometry (Å, °)

**Table:**

*D*—H···*A*	*D*—H	H···*A*	*D*···*A*	*D*—H···*A*
N1A—H1NA···O1A	0.87 (3)	1.89 (2)	2.596 (2)	137 (2)
N1B—H1NB···O1B	0.78 (2)	1.99 (2)	2.600 (2)	134 (2)
C9A—H9AA···O2A^i^	0.95	2.41	3.084 (2)	127
C3B—H3BA···O1B^ii^	0.95	2.51	3.199 (2)	130
C9B—H9BA···O2B^ii^	0.95	2.39	3.174 (3)	139
C17A—H17A···O3A^iii^	0.95	2.59	3.498 (3)	161

Symmetry codes: (i) −*x*+3/2, *y*−1/2, −*z*+1/2; (ii) −*x*+1/2, *y*+1/2, −*z*+1/2; (iii) *x*+3/2, −*y*−1/2, *z*+1/2.
